# The Endoscopic Management of Zenker’s Diverticulum: A Comprehensive Review

**DOI:** 10.3390/diagnostics14192155

**Published:** 2024-09-27

**Authors:** Giuseppe Dell’Anna, Ernesto Fasulo, Jacopo Fanizza, Rukaia Barà, Edoardo Vespa, Alberto Barchi, Paolo Cecinato, Lorenzo Fuccio, Vito Annese, Alberto Malesci, Francesco Azzolini, Silvio Danese, Francesco Vito Mandarino

**Affiliations:** 1 Gastroenterology and Gastrointestinal Endoscopy Division, IRCCS San Raffaele Institute, Via Olgettina 60, 20132 Milan, Italy; dellanna.giuseppe@hsr.it (G.D.); fasulo.ernesto@hsr.it (E.F.); fanizza.jacopo@hsr.it (J.F.); bara.rukaia@hsr.it (R.B.); vespa.edoardo@hsr.it (E.V.); barchi.alberto@hsr.it (A.B.); malesci.alberto@hsr.it (A.M.); azzolini.francesco@hsr.it (F.A.); mandarino.francesco@hsr.it (F.V.M.); 2 Gastroenterology and Gastrointestinal Endoscopy Division, IRCCS Policlinico San Donato, Piazza Edmondo Malan 2, 20097 San Donato Milanese, Italy; vito.annese@grupposandonato.it; 3 Faculty of Medicine and Surgery, Vita-Salute San Raffaele University, Via Olgettina 56, 20132 Milan, Italy; 4 Unit of Gastroenterology, Department of Medical and Surgical Sciences, S. Orsola-Malpighi University Hospital, University of Bologna, Via Massarenti 9, 40138 Bologna, Italy; paolo.cecinato@gmail.com (P.C.); lorenzofuccio@gmail.com (L.F.)

**Keywords:** Zenker’s diverticulum, flexible endoscopic septum division (FESD), Zenker-peroral endoscopic myotomy (Z-POEM), peroral endoscopic septomyotomy (POES), peroral endoscopic diverticulotomy (POED), third-space endoscopy

## Abstract

Zenker’s Diverticulum (ZD) is the most common hypopharyngeal diverticulum; however, it is often underdiagnosed. It results from the herniation of the mucosa and submucosa through Killian’s Triangle. Dysphagia is the primary symptom, occurring in 80–90% of cases. The primary goal of treatment is to transect the cricopharyngeal muscle (CM) and connect the ZD cavity to the esophageal lumen. Traditional treatments include surgical open transcervical diverticulectomy and CM septomyotomy, using rigid or flexible endoscopes. However, surgery is burdened by technical difficulties and not negligible rates of adverse events (AEs). For this reason, endoscopic techniques for ZD treatment have gained traction in recent years. Flexible endoscopic septum division (FESD), introduced nearly 20 years ago, involves a full-thickness incision of the diverticular septum. The advent of third-space endoscopy has led to the application of these techniques to ZD treatment as well. Zenker-POEM (Z-POEM) and, subsequently, Per Oral Endoscopic Septomyotomy (POES) have been developed. Hybrid techniques, such as Peroral Endoscopic Diverticulotomy (POED) and tunneling-free methods, represent additional ZD treatment options. This review outlines the armamentarium of ZD endoscopic management, summarizing the characteristics of these techniques, their benefits and limitations, and highlighting future research directions.

## 1. Introduction

Zenker’s Diverticulum (ZD) is the most common hypopharyngeal diverticulum. It consists of a pulsion diverticulum resulting from the herniation of the mucosa and submucosa of the posterior pharyngeal wall through Killian’s Triangle (KT), a locus minoris resistentiae between the oblique (thyropharyngeal muscle) and transverse fibers (cricopharyngeal muscle (CM)) of the lower pharyngeal constrictor muscle [1,2]. The pathogenesis of ZD is not entirely clear but appears to be related to increased intraluminal pressure at KT, often due to altered relaxation of the upper esophageal sphincter (UES) and the CM. Over time, this process favors the formation of the diverticulum [3]. 

The estimated prevalence of ZD is 0.01–0.11% [4], but this is likely underestimated because asymptomatic patients are unlikely to receive a diagnosis [4]. Epidemiological data indicate that ZD is frequently diagnosed in predominantly male patients between the ages of 70 and 80 [4]. 

Dysphagia is the most common symptom of ZD (80–90%), but patients may also experience regurgitation of undigested food, halitosis, cough, the sensation of a lump in the throat, and, in severe cases, aspiration pneumonia. Esophagogastroduodenoscopy (EGD) and esophagus X-ray with oral contrast medium have traditionally been used for the diagnosis. Historically, ZD has been treated with surgical diverticulectomy performed either through open surgery or using a rigid endoscope. Given the technical issues related to restricted neck mobility and the invasiveness of surgical treatment, endoscopic treatment techniques for ZD have gained traction in recent years [5]. 

Flexible endoscopic septum division (FESD) was the first endoscopic technique introduced 20 years ago [6]. The development of third-space endoscopy has enabled the application of these principles to the management of ZD [7]. Zenker Per Oral Esophageal Myotomy (Z-POEM) was first introduced by Hernandez Mondragon et al. in 2018 [8]. A variant, Per Oral Endoscopic Septomyotomy (POES), was subsequently developed [9]. Several hybrid techniques, such as Peroral Endoscopic Diverticulotomy (POED) and tunneling-free techniques, are further endoscopic treatment options for ZD [10,11,12,13,14,15,16]. 

This comprehensive review outlines the endoscopic armamentarium for ZD and analyzes these procedures’ technical steps, advantages, and limitations. We focus on highlighting a treatment algorithm, which is currently lacking, and future research directions in this field.

## 2. Materials and Methods

In our review, we searched PubMed, PubMed Central (PMC), and Medline including only English-written articles. We implemented a comprehensive search strategy using the following search string: (zenker’s diverticulum [Title/Abstract]) OR (hypopharyngeal diverticulum [Title/Abstract])) AND (endoscopic treatment [Title/Abstract])) OR (flexible endoscopic [Title/Abstract])) OR (POEM [Title/Abstract])) OR (endoscopic septotomy [Title/Abstract]). Additionally, we manually searched the references of the included studies and relevant reviews to identify any additional suitable publications.

## 3. Clinical Assessment

Clinical assessment is pivotal because ZD treatment is indicated only in the presence of symptoms [5]. Treatment for symptomatic ZD aims to transect the CM to create a connection between the ZD cavity and the esophageal lumen. This procedure alleviates the obstruction and helps restore normal swallowing function. 

Although there is not a universally accepted and specific symptom score for the clinical evaluation of ZD patients, different scores have been used (Table 1). 

The Dakkak and Bennett (DB) numerical score primarily focuses on dysphagia, while the Eckardt score, which was originally developed for the evaluation of achalasia, is a more comprehensive tool that assesses dysphagia, regurgitation, retrosternal pain, and weight loss [17]. The Kothari-Haber (KH) score allows for an even broader evaluation of patients, including other symptoms such as cough and hoarseness [18,19]. Both the Eckardt and KH scores have been considered and adapted for the assessment of ZD, with the KH score being the only one currently validated for this condition [20].

As dysphagia represents the most frequent symptom, along with EGD and esophagogram, there is increasing evidence supporting the use of the functional lumen imaging probe (FLIP) for impedance planimetry of the UES in the diagnostic and peri-operative work-up [21]. In a recent study by Che SYW, which included 54 patients, a mean distension index (DI) increase of 2.0 ± 1.7 mm^2^/mmH (*p* < 0.001) was found after Z-POEM [21]. When the authors stratified patients with good (*n* = 23) and poor (*n* = 7) clinical responses, they found that the first group had a significantly higher increase in Distensibility Index (DI) compared to the poor clinical response group (1.6 ± 1.1 vs. 3.3 ± 2.9 mm^2^/mmHg; *p* = 0.037) [21].

**Table 1:** Main symptom scores used in the literature to assess clinical success in cases of endoscopic treatment of Zenker’s Diverticulum.

	Dysphagia		Regurgitation		Weight Loss		Retrosternal Pain		Halitosis		Cough		Hoarseness		Pneumonia		Max Score
[18]	No	0															4
	Solids	1															
	Semisolids	2															
	Solids	3															
	Aphagia	4															
[17]	No	0	No	0	None	0	No	0									12
	Occasional	1	Occasional	1	<5 kg	1	Occasional	1									
	Daily	2	Daily	2	5–10 kg	2	Daily	2									
	Each meal	3	Each meal	3	>10 kg	3	Each meal	3									
[20]	No	0	No	0	None	0			No	0	No	0	No	0	No	0	16
	Solids	1	Occasional	1	0.45–4.54 kg	1			Occasional	1	Occasional	1	Occasional	1	Yes	2	
	Semisolids	2	Daily	2	4.99–9.07 kg	2			Daily	2	Daily	2	Daily	2			
	Solids	3			>9.07 kg	3											
[22]	No	0	No	0	None						0						10
	Solids	1	>1/week and <1/day	1	Recurrent chest infections OR unintentional weight loss >5 kg over last 3 months						1						
	Semisolids	2	≥1/day	2	Recurrent chest infections and unintentional weight loss						2						
	Solids	3	Each meal	3													
	Difficulty swallowing saliva	4	Choking or coughing	4													

## 4. Zenker’s Diverticulum Surgical Treatment

Before the advent of endoscopic treatments, surgery was the only viable option for treating ZD. The available surgical techniques included open surgery (or transcervical diverticulectomy) and septotomy assisted by a rigid diverticuloscope [23,24,25].

Open diverticulectomy involves the following steps. The patient is positioned supine with the neck hyperextended and slightly rotated to the right. An incision is made to the left of the sternocleidomastoid muscle, after which the diverticular pouch is identified. The diverticulum is then carefully dissected from the surrounding connective tissue, exposing its neck. A myotomy of the CM, extending 5 cm towards the cervical esophagus, is then performed. At this point, there are three possible options: (1) excision of the ZD (e.g., with a stapler or manually), (2) suturing the ZD cranially to the paravertebral fascia or the posterior pharyngeal wall, or (3) inverting the ZD into the esophageal lumen [24].

Open diverticulectomy with CM myotomy has been found to lead to the achievement of clinical efficacy rates ranging from 80% to 100%. However, it is associated with adverse events (AEs), even major ones, in up to 11% cases, including vocal cord paralysis (3%), mediastinitis, pharyngo-cutaneous fistulas (3%), wound infection (2%), and esophageal strictures [4]. Additionally, the drainage catheter, which is usually left in place for 24–48 h post-operation, can cause potential discomfort for the patient.

The recurrence rate, defined as the recurrence of symptoms often related to incomplete or ineffective myotomy, ranges from 5% to 10% [4].

Septotomy using a rigid endoscope is another surgical option [26,27]. During the procedure, the patient is positioned supine with the neck hyperextended. The process involves resecting the diverticular septum with a stapler after positioning a rigid diverticuloscope to obtain an optimal view of the ZD. In recent years, electrocautery, carbon dioxide lasers, or harmonic scalpel devices have been proposed as alternatives to the stapler [28]. However, the procedure is often limited by suboptimal technical success (TS), primarily due to the need for neck hyperextension and adequate visualization of the diverticulum. These factors are the main causes of technical failure, which can necessitate a switch to open surgery in about 5% to 7% of cases [29,30,31]. The clinical success (CS) rate was found to range from 63 to 100%, with an adverse event (AE) rate of 10% [4]. The most common AEs include bleeding, dental damage, esophageal mucosal injuries, leaks, and perforations [29,30,31].

In a meta-analysis conducted by Bhatt N. K. et al., which included nine studies with 903 patients, the efficacy and safety of transcervical diverticulectomy, stapler-assisted diverticulotomy, and laser-assisted diverticulotomy were compared. Open diverticulectomy resulted in a lower rate of persistent/recurrent symptoms compared to the other two procedures (odds ratio [OR], 0.20; 95%CI, 0.04–0.91). In the comparison between laser-assisted and stapler-assisted diverticulotomy, no significant difference in recurrence rates was found (OR, 0.83; 95%CI, 0.43–1.60) [23].

## 5. Endoscopic Management

Flexible endoscopic approaches have been developed over the past few decades as safe and effective alternatives to surgery for ZD treatment [7,32]. These endoscopic techniques aim to address the challenges associated with traditional surgical methods, such as the invasiveness of open surgery and the technical difficulties posed by rigid endoscopes. Since ZD predominantly occurs in the elderly, who often have multiple comorbidities and a higher surgical risk, a minimally invasive endoscopic approach offers significant advantages [3,33].

### 5.1. Flexible Endoscopic Septum Division (FESD)

**Figure 1:**
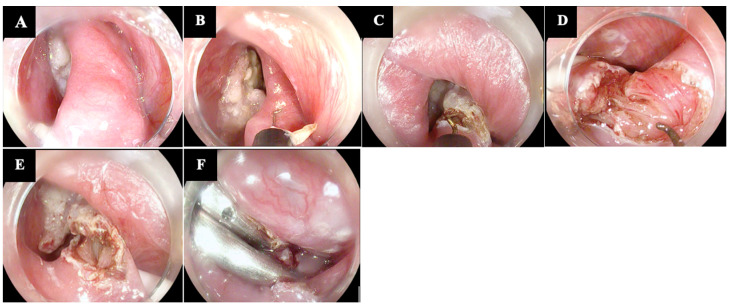
Technical steps of flexible endoscopic septum division (FESD). (**A**) Visualization of the septum that separates the Zenker’s Diverticulum (ZD) from the esophagus and which contains the cricopharyngeal muscle (CM); a transparent hood is usually placed on the tip of the endoscope to stabilize positioning. (**B**,**C**) Septotomy performed by a J-shaped knife. (**D**,**E**) Final stages of the septotomy with exposure of the CM fibers. (**F**) Placement of through-the-scope clips at the base of the diverticulum at the end of the septotomy. The copyright of the image belongs to the authors.

Mulder et al. and Ishioka et al. first described FESD over 20 years ago. FESD operates on the same principles as RES, as the septum separating the ZD from the esophagus contains the CM, and dividing this septum endoscopically leads to a CM myotomy (Figure 1) [3].

FESD is performed with the patient in the left lateral decubitus position. The procedure can be safely performed in an inpatient or outpatient setting and does not strictly require general anesthesia. Additionally, it does not necessitate neck hyperextension, which is required for septotomy guided by a rigid endoscope [34]. No prophylactic antibiotic therapy is needed [35]. The technique can be enhanced with accessories such as a soft diverticuloscope and an overtube to improve septum exposure. A hood is usually placed on the tip of the endoscope to stabilize positioning [36].

Before starting FESD, a nasogastric/orogastric tube or a guidewire is always placed in the esophageal lumen as a landmark during the septotomy [37]. This enhances the safety of the procedure by ensuring accurate identification and protection of the esophageal wall. A submucosal injection is rarely used before the incision [38,39]. The primary cutting technique is performed with a J-shaped knife or triangle-type knife using either mixed or pure coagulation current [40]. Other less common cutting methods include monopolar forceps and argon plasma coagulation [30]. The myotomy must be carefully balanced to avoid being too short while also not extending beyond the inferior border of the diverticulum, which could cause mediastinal perforations [41]. One or more through-the-scope clips (TTSCs) can be placed at the base of the diverticulum at the end of the CM myotomy [33].

The single-incision method, which entails cutting along the midline of the CM, is the most used technique [42]. Another proposed technique is the double-incision method, which allows for the dissection of a larger septum and involves two incisions spaced 1 cm apart. The central septum is then resected using a snare [43].

FESD has demonstrated high clinical efficacy, with CS rates ranging from 90% to 100% across multiple studies [6,44,45,46].

In a meta-analysis by Ishaq et al., including 20 studies with 813 patients with ZD treated by FESD, the CS rate was 91% [47].

In a meta-analysis by Deepanshu et al., which included 997 patients, the outcomes of FESD performed using different modalities were compared: standard FESD (342 patients), FESD with a soft diverticuloscope (FESD-D) (487 patients), and FESD with a distal cap (FESD-C) (126 patients). FESD showed an overall TS rate of about 99%. The clinical success (CS) rates were 86.8% for the FESD-D group, 75.4% for the FESD-C group, and 94.0% for the standard FESD group [6].

FESD has proven to be a safe technique with a low recurrence rate. In the meta-analysis by Ishaq et al., the overall AE rate was 11% (813 patients in total). Among the 84 AEs reported were bleeding (*n* = 26), pneumonia (*n* = 3), fever (*n* = 15), subcutaneous emphysema (*n* = 16), and perforation (*n* = 28), which were all treated conservatively with antibiotic administration, and a neck abscess (*n* = 1) that required incision and drainage. These AEs did not appear to be associated with clinical risk factors (such as ZD size or prior treatments) or technical aspects, including the type of sedation or the cutting device used [47]. In the study by Deepanshu J. et al., the rates of bleeding and perforation were 6.6% and 5.3%, respectively [6].

Ishaq et al. reported an overall recurrence rate of 11% (95%CI, 8–15%). In the meta-analysis by Deepanshu et al., the FESD-D group showed a higher clinical recurrence rate compared to the FESD-C and standard FESD group (16.5% vs. 9.5% vs. 11.1%, respectively) [6].

Long-term outcomes (about 6 months or more) after FESD have been evaluated in several studies.

Brueckner J. et al. reported their experience with 46 patients treated for ZD using FESD [48]. The DB score was used to assess clinical status. Initially (at the time of hospital discharge) the CS rate was 100%, but 30% of patients experienced symptom relapse after an average of 4.4 months (range 1–40 months). These patients required an average of 1.39 additional FESD procedures per patient, leading to a total 95% clinical success. Patients with recurrence had more severe pre-procedural symptoms, such as vomiting (2.13% vs. 0.92%; *p* < 0.05) and insomnia (1.65% vs. 1.08%; not significant), and a higher median DB score, although this was not significant (2.25 vs. 1.59) [48].

Ishaq S. et al. conducted a study with 65 patients, including those newly diagnosed with ZD (*n* = 36) and those with recurrent ZD symptoms after rigid endoscopic septotomy (*n* = 29), treated with FESD. The Dysphagia–Regurgitation–Complication (DRC) score was used for clinical evaluation [44]. After FESD, over an average follow-up of 19 months, symptom remission and improvement rates were 75.4% and 92.7%, respectively. The rate of patients requiring repeated FESD was 5.6% in the treatment-naive group and 34.5% in the recurrent group (*p* = 0.003). At 48 months, recurrence was higher in patients with recurrent ZD (84.7%) than in treatment-naive patients (10.7%) [44].

The most recent study with long-term data on FESD is a prospective single-center study by Repici A. et al., which included 256 patients treated with FESD [49]. After achieving a CS rate of 96.1%, symptom recurrence occurred in 31.3% (80/256) of patients at a mean time of 9 ± 3 months, as defined by the DB score. Almost all these patients (95%) were treated with a second FESD. After a median follow-up of 5.5 years, 95.3% of patients did not experience symptom relapse [49].

### 5.2. Zenker-Peroral Endoscopic Myotomy (Z-POEM)

A new approach using a flexible endoscope was introduced in 2016, inspired by endoscopic peroral myotomy (POEM). Initially named submucosal tunneled division of the endoscopic septum (STESD), it was later rebranded as Z-POEM [8]. The potential benefit of Z-POEM lies in its ability to transect the entire muscular septum within a submucosal tunnel, thereby more effectively preserving mucosal integrity [50].

The procedure is performed with the patient in the left lateral decubitus position. It begins with a submucosal injection followed by a longitudinal mucosal incision approximately 3 cm proximal to the septum, which serves as the entry point into the tunnel. A submucosal tunnel is then created along both sides of the septum, extending 1–3 cm distal to the base of the diverticulum. Once the septum is fully exposed, a myotomy is performed down to the base of the ZD and into the normal esophageal muscle. The mucosal entry is then closed with TTSCs [3].

The outcomes of Z-POEM have been evaluated in several studies and meta-analyses.

In a multicenter international retrospective study by Yang et al. involving 75 patients undergoing Z-POEM, the TS rate was 97.3%, and CS, defined as the complete or nearly complete resolution of dysphagia, was achieved in 92% of patients. The mean procedure time was 52.4 min (SD 2.9) [50]. In a more recent multicenter retrospective study involving 24 patients with ZD who underwent Z-POEM across five countries, the TS rate was 100%, and CS, defined as the complete or near-complete resolution of symptoms, was achieved in 95.8% of cases. The median procedure time was 61 min [51].

Budnicka A. et al. conducted a retrospective multicenter study at three Polish tertiary referral centers, including 22 patients with symptomatic ZD treated with Z-POEM. The study reported a TS rate of 100% and a CS rate of 90.9%, defined as the complete resolution of dysphagia and nearly complete resolution of associated symptoms. The mean procedure time was 48.8 min [52]

In another multicenter cohort study by Zhang L. Y. et al., involving 11 centers and 83 patients, Z-POEM achieved TS in 98.8% of patients, with a mean procedure time of 48.7 min [52]. CS, defined as achieving a DB score ≤ 1, was reported in 85.5% of cases [52].

In the recent international multicenter study conducted by Steinway S. et al., which involved 89 patients undergoing Z-POEM [53], TS was 97.8% and CS, defined as improvement in the modified DB Score to ≤1 if the initial score was >1, and to 0 if the initial score was 1, was 94% [54].

In a recent meta-analysis by Zhang H. et al. including 11 studies involving 357 patients who underwent Z-POEM, the overall TS rate was 96.3% (95%CI 93.6–97.9%; I^2^ = 0%, Q test *p*-value = 0.990), and the pooled rate of total CS was 93.0% (95%CI 89.4–95.4%; I^2^ = 0%, Q test *p*-value = 0.988) [55].

Z-POEM demonstrated similar safety outcomes compared to FESD.

Yang et al. reported an AE rate of 6.7%, including one mild hemorrhage, managed conservatively, and four perforations (one requiring intensive care unit [ICU] monitoring, two treated endoscopically with cyanoacrylate glue, and one closed with clips) [50]. Budnicka A. et al. reported two cases of asymptomatic subcutaneous emphysema of the neck and one case of extensive subcutaneous and intramuscular emphysema with laryngeal edema, which required prolonged intubation and ICU monitoring [54]. Zhang L. Y. et al. reported post-procedural AEs in 4.8% of cases, including two leaks and two post-procedural infections [52]. In the study by Steinway et al., AEs occurred in eight patients (8.9%). These included three cases (3.4%) of perforations, with one managed conservatively and the other two endoscopically; three cases (3.4%) of moderate bleeding, all managed endoscopically; and one case each of aspiration pneumonia and pneumomediastinum, both mild and treated conservatively [53]. The pooled occurrence of AEs in the meta-analysis of Zhang H. et al. was 12.4% (95%CI 9.1–16.7%; I^2^ = 0%, Q test *p*-value = 0.507), with the most reported being bleeding, perforations, and subcutaneous emphysema [55].

Some evidence has confirmed the long-term efficacy of Z-POEM.

In the study by Steinway et al., a 2-year recurrence rate of 6.7% was reported [53]. In the meta-analysis by Zhang H. et al., which included data from 7 out of 11 studies with up to 24 months of follow-up, the pooled clinical recurrence rate for Z-POEM was found to be 11.2% (95%CI 7.6–16.2%; I^2^ = 0%, Q test *p* = 0.475) [55]. In the meta-analysis by Facciorusso A. et al., which included 12 studies and 300 patients, 1-year efficacy was maintained in 90% of patients (86.4–97.4%) and in 89.6% (82.2–96.9%) of cases 2 years after Z-POEM [56].

### 5.3. Peroral Endoscopic Septotomy (POES)

Managing ZD with a short septum (≤20 mm) presents significant challenges for endoscopists. The limited anatomical space can make it difficult to place the necessary devices. Additionally, the initial mucosal incision, typically performed 3 cm proximal to the septum, can be complicated by muscle spasms and anatomical constraints, which may hinder the proper opening and placement of clips to close the mucosal incision [3].

**Figure 2:**
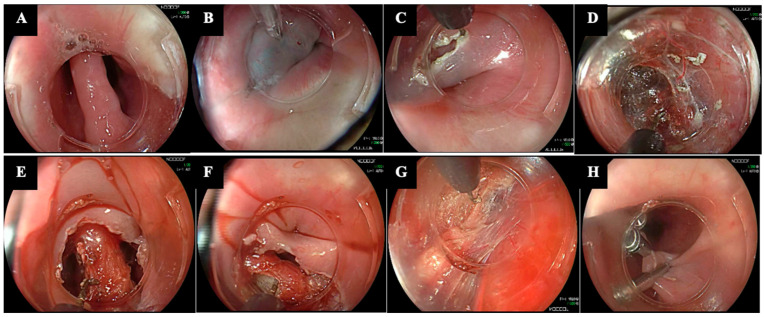
Technical steps of Peroral Endoscopic Septotomy (POES). (**A**) Visualization of the Zenker’s Diverticulum septum by placing a transparent distal hood. (**B**) Submucosal injection is performed just above the septum. (**C**) The mucosal entry is performed directly on the septum with a J-shaped knife. (**D**) Submucosal tunneling is created along both sides of the septum, terminating from 1 to 3 cm distal to the base of the ZD. (**E**–**H**) Exposure of the cricopharyngeal muscle and subsequent myotomy down to the base of the ZD and into the esophageal muscle. (**H**) Final closure of the mucosal defect with through-the-scope clips. The copyright of the image belongs to the authors.

To address these issues, Repici A. et al. proposed an alternative submucosal approach called Peroral Endoscopic Septotomy (POES) (Figure 2) [9].

The key feature of POES lies in performing the mucosal entry directly on the septum, rather than 3 cm above it. The remaining procedural steps are the same as in Z-POEM.

In the initial experience, the POES technique achieved a 100% TS rate for complete septal myotomy and led to 95% CS, defined as complete or near-complete symptom resolution (DB score of 0 or 1). No intra- or post-procedural AEs were reported. The mean procedure time was only 13.8 min [9].

In the study by Mittal C. et al., which involved 24 ZD patients undergoing POES [56], TS was 95.8% and CS 90.9%. AEs were reported in 16.7% of cases, with the most common being perforation at the site of septotomy. The post-POES recurrence rate was 8.3% (2/24), and these cases were successfully treated by repeating the procedure [56].

In a retrospective single-center study including 19 patients, POES led to 94.7% TS. CS, defined as a dysphagia DB score of 0 from an initial score of 1, or a score of 1 or 0 from an initial score of 2 or higher, was 89.5%. Symptom recurrence was observed in two patients (11.7%) at 6 months and 24 months. The average procedure duration was 27 min. AEs were observed in 3 out of 19 patients, including a retropharyngeal abscess and two perforations [57].

In the meta-analysis by Spadaccini M. et al., which included three studies and 63 patients undergoing POES, the technique was associated with a TS rate of 96.5% (95%CI, 92.0–100.0%; I^2^ = 0%; *p* = 0.879) and a CS rate of 91.1% (95%CI, 84.1–98.0%; I^2^ = 0%; *p* = 0.420). The mean procedural time was 20.4 ± 9.3 min [58].

### 5.4. Hybrid Techniques

In the last 5 years, several hybrid endoscopic techniques have been proposed (Table 2) [10,11,12,13,14,59].

These approaches integrate the principles of classical FESD with those of third-space endoscopic procedures (Z-POEM, POES) [3,19]. The aim is to combine the benefits of both methods, enhancing safety and efficacy while minimizing the limitations associated with each technique.

#### 5.4.1. Submucosal Injection Techniques

In 2018, Pugliese F. et al. proposed the first hybrid technique, called Peroral Endoscopic Diverticulotomy (POED) [60]. During the myotomy, a submucosal injection of saline, adrenaline, and indigo carmine is performed on both the diverticular and esophageal sides. This helps to better expose and delineate the muscle and guide the myotomy. In the initial experience of five patients, POED achieved 100% TS and CS rates, with an average procedure duration of 20 min. No major AEs were reported [10].

In 2020, Hu H. et al. reported a case of a ZD patient treated by an alternative FESD technique, called Transverse Incision and Longitudinal Septostomy (TILS) [14]. The main difference from FESD is the transverse incision, as opposed to the longitudinal incision through the mucosa and muscle of the septum. This transverse approach offers a larger operating space and better visibility, enabling a more complete and accurate septotomy [14].

**Figure 3:**
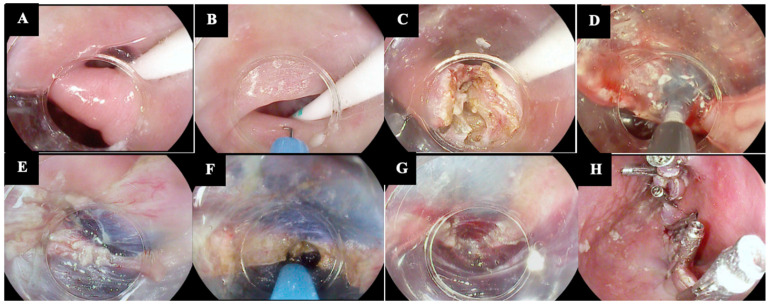
Technical steps of Submucosal INjection Guided septomyotomy (SING technique). (**A**) Visualization of the septum by using a transparent distal hood and by inserting a nasogastric/orogastric tube into the esophageal lumen. (**B**,**C**) A full-thickness septotomy with a J-shaped knife is performed for about two-thirds of the septum’s length. (**D**–**G**) A complete CM myotomy is performed, extending approximately 10 mm into the esophageal muscle, with continuous submucosal injection on both sides to precisely identify the muscle fibers. (**H**) Placement of through-the-scope clips to close the mucosal defect. The copyright of the image belongs to the authors.

Our group recently proposed a new hybrid technique named Submucosal INjection Guided septomyotomy (SING technique) (Figure 3) [13,16].

The SING technique, like POED, involves a classic septotomy guided by a submucosal injection, which helps to better delineate and expose the muscular layer and guide the complete myotomy, without the need for bilateral submucosal tunneling. The submucosal injection is performed midway through the septotomy. The main difference to POED is that the SING technique does not use a soft diverticuloscope; it relies only on a distal cap. In the initial experience involving nine patients, the technique was effective in all cases, with no AEs and no recurrence after a median follow-up of 217 days [13,16].

#### 5.4.2. Submucosal Tunneling Techniques

One of the main issues associated with Z-POEM, particularly for large ZDs, is the creation of a mucosal flap, which could paradoxically contribute to post-intervention dysphagia [15,59]. To address this, Zhang L. Y. et al. proposed an alternative Z-POEM technique that includes a mucosotomy at the end of the myotomy. The mucosal flap is stabilized by placing one TTSC at each end and then a stag beetle knife is used to transect the mucosal flap down to the most inferior aspect of the diverticulum [60]. In a series of five patients, CS was achieved in all patients. No AEs and no symptom recurrence during the follow-up period were reported [59].

Mavrogenis G. et al. proposed another modified version of Z-POEM, called single-tunnel Z-POEM [12]. It involves the creation of a single submucosal tunnel instead of two. The main advantage is the reduction in procedural time. However, the main potential drawback is the risk of unintentional injury to the esophageal mucosa if the submucosal cushion is inadequate [12].

Estevinho M. M. et al. recently published a case series of four patients with ZD treated by a new technique called tunneling-free Z-POEM, also known as R-POES (Readily POES) [11]. The rationale behind the development of R-POES is that submucosal tunneling can be challenging and may cause additional damage, especially to the esophageal mucosa. R-POES starts with a mucosal injection over the septum followed by a mucosal incision. Next, the submucosa are dissected, and once the muscular septum is exposed, a full-thickness septotomy is performed. Differently from Z-POEM, tunneling is not performed; instead, the submucosa on both sides are only injected. At the end of the septotomy, the mucosal incision is secured with TTSCs. In the initial experience, R-POES was effective in all patients, with no AEs. The procedure’s mean duration was 36 ± 14 min [11].

## 6. Comparative Data

Although several endoscopic techniques have been proposed over the last few decades for the management of ZD, only a few comparative studies are available, both between surgical procedures and endoscopic techniques and among the various endoscopic procedures (Table 3).

### 6.1. Surgery vs. Endoscopy

Rudler F. et al. recently published a large retrospective study including a total of 144 patients, comparing the safety and efficacy of open surgery (*n* = 48), rigid endoscopic septotomy (RES) (*n* = 52), and FESD (*n* = 44) [29]. The DB score was used for clinical evaluation. RES showed a lower TS rate (88%) compared to both FESD (98%) and open surgery (100%) (*p* = 0.014). Open surgery was associated with a longer median procedural time (85 min) compared to RES (45 min) and FESD (47 min) (*p* < 0.001) and a longer hospital stay (6 days for open surgery vs. 3 days for both RES and FESD; *p* < 0.001). Both surgical approaches showed similar clinical efficacy compared to FESD, but open surgery had a significantly higher CS rate (97%) compared to RES (79%) (*p* = 0.016). No differences were found in the AE rate between the three groups [29].

According to a systematic review and meta-analysis of 11 studies, conducted by Albers et al. comparing endoscopic versus surgical treatment for ZD in 596 patients, the endoscopic approach resulted in a shorter length of procedure [SMD − 78.06 (95%CI − 90.63, −65.48) *p* < 0.00001], length of hospitalization [SMD − 3.72 (95%CI − 4.49, −2.95) *p* < 0.00001], time to diet introduction [SMD − 4.30 (95%CI − 5.18, −3.42), *p* < 0.00001], and complication rate [SMD − 0.09 (95%CI 0.03, 0.43), *p* = 0.02] compared to surgery [27]. However, the surgical approach was associated with a statistically significant reduction in symptom recurrence rates [SMD 0.08 (95%CI 0.03, 0.13) *p* = 0.002] compared to endoscopy.

The most recent meta-analysis comparing open surgery, FESD, and RES included eight retrospective comparative studies with a total of 1281 patients: 336 in the open surgery group, 453 in the RES group, and 492 in the FESD group [63]. The authors did not find differences in technical success (TS) [risk difference (RD), 0.07 (95%CI − 0.03 to 0.16); *p* = 0.18], clinical success (CS) [RD, 0.07 (95%CI − 0.05 to 0.19); *p* = 0.26], adverse events (AEs) [RD, −0.03 (95%CI − 0.13 to 0.07); *p* = 0.052], and procedure time [mean difference, −10.03 (95%CI − 26.93 to 6.88); *p* = 0.24]. However, FESD showed a lower hospital stay in days compared with surgery [mean difference, −1.98 (95%CI − 3.56 to −0.40); *p* = 0.001] [63].

### 6.2. Endoscopy vs. Endoscopy

Al Ghamdi S. S. et al. conducted the largest multicenter study available, comparing 245 patients with ZD treated by Z-POEM (*n* = 119), FESD (*n* = 86), or RES (*n* = 40) [61]. TS rates were similar across the groups (95% for Z-POEM, 95.3% for FESD, and 87.5% for RES, *p* = 0.18). Notably, the FESD group had a significantly shorter procedural time compared to both Z-POEM and RES (33.72 min vs. 46.13 min vs. 54.03 min, respectively, *p* < 0.001). The CS, defined as a decrease in the DB score to ≤1, was similar between the three groups: 92.7% (102/110) in the Z-POEM group, 89.2% (33/37) in the RES group, and 86.7% (65/75) in the FESD group (*p* = 0.26) [61]. The RES group, however, had a higher rate of AEs (30.0%) compared to the Z-POEM (16.8%) and FESD groups (2.3%) (*p* < 0.05). Symptom recurrence rates did not show statistically significant differences among the groups: 14.7% in the Z-POEM group (mean follow-up of 282.04 days), 9.2% in the FESD group (mean follow-up of 262 days), and 9.1% in the RES group (mean follow-up of 125 days) [61].

A recent study by Swei E. et al. [27] compared FESD and Z-POEM in 28 patients, with 13 undergoing Z-POEM and 15 receiving FESD. The median procedural times were similar: 43.9 min for Z-POEM and 60.2 min for FESD. Both groups achieved a TS rate of 100%. While the Z-POEM group had a higher CS rate (100%) compared to the FESD group (86.7%), the difference was not statistically significant (*p* = 0.18) [62].

Klingler M. J. et al. conducted a retrospective study comparing FESD (*n* = 7) with a new submucosal technique called MIMI (*n* = 19) (see Section 5.3 for the technique description) [57]. CS was similar between the two techniques (89.5% in the MIMI group and 100% in the FESD group, *p* = 0.101). Similarly, there were no significant differences in recurrence rates (11.7% for MIMI vs. 42.9% for FESD; *p* = 0.096) [57].

Submucosal tunneling techniques for ZD management were also evaluated in a meta-analysis by Spadaccini M. et al., which compared Z-POEM (six studies, 133 patients) and POES (three studies, 63 patients) [59]. The TS rates were nearly identical, with 97.1% for Z-POEM and 96.5% for POES. CS rates were similarly close, with 94.1% for Z-POEM and 91.1% for POES [58].

Mittal C. et al. conducted a retrospective study comparing FESD (137 patients) and POES (24 patients) [58]. TS was similar between the two techniques (97.1% for FESD vs. 95.8% for POES, *p* = 0.56). Although the CS rate was higher for POES compared to FESD, the difference was not statistically significant (90.9% vs. 75.2%, respectively, *p* = 0.16). The AE rate was also higher in the POES group, though it did not reach statistical significance (16.7% for POES vs. 6.6% for FESD, *p* = 0.11) [56].

## 7. Future Perspectives

The endoscopic management of ZD has evolved significantly in recent years. The first technique introduced, FESD, has been complemented over the past 20 years by the application of advanced third-space endoscopic procedures to the treatment of ZD, with techniques such as Z-POEM and POES. Additionally, hybrid techniques have emerged, aiming to combine the benefits of various approaches while minimizing their limitations.

While surgery has traditionally been the standard for ZD, endoscopic techniques now provide comparable, if not superior, clinical outcomes with a significantly lower risk of AEs. FESD has demonstrated higher technical feasibility and a better safety profile compared to RES, which is severely limited by the need for neck hyperextension for rigid diverticuloscope placement and open diverticulectomy, which was characterized by a longer procedural time and hospital stay [29,61,63]. Another aspect that emerges is the potential futility of using a soft diverticuloscope during the FESD procedure [6]. Its placement and use could be associated with increased procedural time and intra-operative AEs, without a significant benefit in terms of technical and clinical efficacy [6]. Some long-term studies have reported a recurrence rate of about 30% associated with FESD [48,49]. However, these data could be attributed to the fact that FESD was the first introduced endoscopic technique and is likely the most widely used, leading to more long-term data available for this technique compared to others (e.g., Z-POEM, POES). Additionally, the lack of a standardized clinical evaluation of patients may have contributed to the misinterpretation of symptom assessment. Although submucosal techniques, like Z-POEM and POES, have been proposed for their benefits of more precise myotomy and reduced risk of perforation, the available evidence has shown that these techniques are associated with a higher rate of AEs and longer procedural times, with the same clinical efficacy when compared to FESD [61,62]. FESD, which has been the gold standard for years, now appears to be the foundation for the future, with hybrid variants like our SING technique representing the next evolution—a faster, more effective, and safer approach enhanced by the experience gained in submucosal endoscopy.

Despite the significant progress made in the field, several aspects of the endoscopic treatment of ZD still require further research and development, which we will attempt to summarize in this section.

### 7.1. Terminology and Abbreviations

The terminology and acronyms of techniques used vary across studies, leading to potential confusion and hindering standardization efforts [7]. Establishing a unified terminology, ideally supported by international society guidelines, is essential to harmonize research efforts and mitigate confusion. This is especially crucial considering the various proposed hybrid techniques, which further complicate the comparison and interpretation of study outcomes.

### 7.2. High-Quality Evidence

One crucial aspect is the need for high-quality, prospective, multicenter studies or even randomized controlled trials that directly compare the different techniques. Such studies would provide more robust evidence to inform clinical decision-making and help identify the most suitable technique for specific patient subgroups. The development of consensus guidelines and expert recommendations based on the best available evidence could help streamline the technical aspects of each procedure, minimize variability, and promote the adoption of best practices. Standardization efforts should encompass various aspects of the procedures, including patient selection criteria, pre-procedural clinical evaluation, technical steps, post-procedural care, and follow-up protocols.

### 7.3. Endoscopic Treatment Algorithm

Developing a personalized approach, where the choice of technique is tailored to the specific needs and characteristics of each patient, is a promising avenue to explore [19]. Future research should focus on identifying the optimal indications for each endoscopic technique based on diverticulum characteristics, such as size and morphology. While current guidelines provide general recommendations for the endoscopic management of this condition, there is a need for more nuanced, evidence-based criteria to guide the selection of the most appropriate technique for individual patients [5]. For instance, certain techniques may be more suitable for smaller diverticula (FESD), while others may be better suited for larger cases of recurrence or complex cases (Z-POEM, POES). However, the decision to perform mucosotomy and in which specific cases remains a debated topic [59,64,65].

### 7.4. Standardization of Pre- and Post-Procedural Management

Another important consideration is the setting in which procedures are performed. While some centers may opt for an inpatient approach, there is growing interest in the feasibility and safety of performing these procedures in an outpatient setting. This shift could be associated with potential reductions in healthcare costs, improved patient convenience, and optimized resource utilization. A large single-center cohort analysis showed that ZD endoscopic treatment could be performed as an outpatient procedure with a 2 h post-procedure observation period [66]. All significant AEs were identified during this observation period, and delayed AEs (2.5% of cases) were effectively managed with supportive care [66]. These findings suggest that appropriately selected patients can be safely managed as outpatients, achieving safety outcomes comparable to those observed in admitted patients.

### 7.5. Validated Symptom Scores

Defining and validating clinically relevant outcome measures is crucial for assessing the effectiveness of endoscopic interventions in the management of ZD [22]. Currently, there is not a universally accepted, ZD-specific symptom score. Several studies have used the DB dysphagia numerical score, a simple tool that primarily focuses on dysphagia severity [18]. While useful, it may not capture the full spectrum of symptoms experienced by patients with ZD. In contrast, the KH score is a validated, ZD-specific symptom score that encompasses a broader range of symptoms (weight loss, dysphagia, regurgitation, halitosis, cough, hoarseness, and a history of pneumonia) [20]. Standardizing the use of validated, ZD-specific symptom scores across studies would enable more meaningful comparisons of different endoscopic techniques and their outcomes [67]. Furthermore, incorporating additional patient-reported outcome (PRO) measures that assess various aspects of quality of life could provide valuable insights into the patients’ perspective and guide treatment decisions [68,69].

### 7.6. Endoscopic Training

Given the rarity of this condition, it is extremely difficult to obtain dedicated training, which is essential to ensure that endoscopists acquire the necessary skills and competencies to perform these advanced procedures safely and effectively. The complexity of techniques like Z-POEM, POES, and hybrid approaches may necessitate structured training curricula and hands-on workshops to help bridge the learning curve and promote the widespread adoption of these techniques. Additionally, the use of simulation-based training and animal models can help develop the required dexterity and confidence before performing these procedures on patients [70].

## 8. Conclusions

In conclusion, the endoscopic management of ZD has seen significant advancements in recent years, with the introduction of novel techniques aimed at enhancing efficacy, safety, and patient outcomes. However, there are still several areas that require improvement, such as the need for high-quality comparative studies, the standardization of techniques, dedicated training programs, and the validation of outcome measures. Addressing these current limitations and knowledge gaps is essential for developing more personalized and effective treatment strategies, ultimately improving the quality of life for patients suffering from this rare but often challenging condition.

## Figures and Tables

**Table 2:** Summary of hybrid techniques in the treatment of Zenker’s Diverticulum (ZD). CM, cricopharyngeal muscle; FESD, flexible endoscopic septum division; POED, Peroral Endoscopic Diverticulotomy; Z-POEM, Zenker peroral endoscopic myotomy; R-POES, Readily Peroral Endoscopic Septotomy; TILS, Transverse Incision and Longitudinal Septostomy; SING, Submucosal INjection Guided septomyotomy.

Technique	Authors, Year of Publication	Study Design	Technical Peculiarities	Pros
POED	Pugliese F. et al., 2020 [10]	Case series (5 patients)	- Soft diverticuloscope - J-shaped knife - Submucosal injection during CM myotomy - No submucosal tunneling - No full-thickness septotomy	- More stable position - Reduced risk of perforation (no submucosal tunneling) - Better muscle fiber exposure - Reduced procedural time
TILS	Hu H. et al., 2020 [14]	Case report	- Transverse septum mucosal incision - Longitudinal septotomy - No submucosal tunneling	- Reduced risk of perforation (no submucosal tunneling) - Larger operating space - Better muscle fiber exposure
SING	Azzolini F. et al., 2024 [13]	Prospective series (9 patients)	- FESD of the first two-thirds of the septum - No submucosal tunneling - Complete myotomy guided by submucosal injection at both sides - Extension of the myotomy to the esophageal muscle layer	- Reduced post-procedural dysphagia - Reduced risk of perforation (no submucosal tunneling) - Better muscle fiber exposure - Reduced procedural time
Z-POEM with mucosotomy	Zhang L. Y. et al., 2021 [59]	Case series (4 patients)	- Residual mucosal flap transection	- Reduced post-procedural dysphagia
Single-tunnel Z-POEM	Mavrogenis G. et al., 2023 [12]	Case report	- Single submucosal tunnel	- Reduced procedural time
Tunneling-free Z-POEM or R-POES	Estevinho M. M. et al., 2023 [11]	Case series (4 patients)	- Mucosal injection over the septum - Submucosal dissection until the muscle is exposed - CM myotomy with submucosal injection at both sides	- Reduced risk of perforation (no submucosal tunneling) - Reduced procedural time

**Table 3:** Comparative studies assessing the main outcomes of the endoscopic treatment of Zenker’s Diverticulum. POES, Peroral Endoscopic Septotomy. NA, not applicable. FESD, flexible endoscopic septum division. Z-POEM, Zenker-peroral endoscopic myotomy. RES, rigid endoscopic septotomy.

Author (Year)	Study Design	Technique (n Patients)	Technical Success (%)	Clinical Success (%)	Procedural Time (min)	Adverse Events (%)	Recurrence Rate (%)
Mittal et al. (2020) [56]	Retrospective	POES (24)	95.8	90.2	NA	16.7	NA
		FESD (137)	97.1	75.2	NA	6.6	NA
Spadaccini et al. (2021) [58]	Meta-analysis	Z-POEM (133)	97.1	94.1	46.4	NA	NA
		POES (63)	96.5	91.1	20.4	NA	NA
Klinger et al. (2021) [57]	Retrospective	POES (19)	94.7	89.5	27.0	15.8	11.7
		FESD (7)	100	100	22.0	28.6	42.9
Al Ghamdi et al. (2022) [61]	Retrospective	Z-POEM (119)	95.0	92.7	46.13 (*p* < 0.001)	16.8 (*p* < 0.05)	14.7
		FESD (86)	95.3	86.7	33.7 (*p* < 0.001)	2.3 (*p* < 0.05)	9.2
		RES (40)	87.5	89.2	54.0	30.0 (*p* < 0.05)	9.1
Swei et al. (2023) [62]	Prospective	Z-POEM (13)	100	100	43.9	0	0
		FESD (15)	100	86.7	60.2	6.7	0.8
Rudler et al. (2023) [29]	Retrospective	OPEN SURGERY (48)	100	97	85 (*p* < 0.001)	10	4 (*p* = 0.003)
		RES (52)	88 (*p* = 0.04)	79 (*p* = 0.016)	45 (*p* < 0.001)	19	26
		FESD (44)	98	90	47 (*p* < 0.001)	13	29 (*p* = 0.001)
Albers et al. (2016) [27]	Meta-analysis	ENDOSCOPY 300	NA	NA	42 (*p* < 0.00001)	9.3 (*p* = 0.02)	13
		SURGERY 296	NA	NA	47	14.8	6.4 (*p* = 0.02)
Cadena Aguirre et al. (2023) [63]	Meta-analysis	OPEN SURGERY (336)	NA	93.9	NA	9.8	NA
		RES (453)	79	69.7	NA	4.7	NA
		FESD (492)	92.9	88	NA	7.4	NA

## Data Availability

No new data were created or analyzed in this study. Data sharing is not applicable to this article.
